# Audience segmentation and messaging approach to gain public support and involvement in coastal social-ecological system management

**DOI:** 10.1038/s41598-026-38402-0

**Published:** 2026-02-03

**Authors:** Takuro Uehara, Takeshi Hidaka, Sawako Tachibana

**Affiliations:** 1 College of Policy Science, Ritsumeikan University, 2-150 Iwakura-Cho, Ibaraki City, Osaka 567-8570 Japan; 2 Faculty of Humanity-oriented Science and Engineering, Kindai University, Iizuka, Fukuoka Japan; 3 Graduate School of Humanity-oriented Science and Engineering, Kindai University, Iizuka, Fukuoka Japan

**Keywords:** Audience segmentation, Coastal management, Messaging, Public involvement/engagement, Social-ecological system, Environmental social sciences, Environmental studies, Geography, Geography, Ocean sciences

## Abstract

Gaining public support and involvement is essential for implementing effective social-ecological system (SES) management. Earlier studies focused on an aggregate level, i.e., measured by average; however, we aimed to address public heterogeneity, determine their support for policy, and identify their involvement in SES management. Accordingly, we applied audience segmentation and a messaging approach in coastal SES management to address oligotrophication in part of the Seto Inland Sea (SIS), Japan. In our experimental study, audience segmentation was applied based on a single-item self-categorization measure regarding public opinion about coastal SES issues and management, and the impact of two messaging conditions on the segments was assessed. Respondents living in coastal SES areas were recruited (N = 1800) and randomly divided into three groups: a control group without messaging and two groups with either “Negative consequences of oligotrophication” or “Collective public involvement” messaging. Four primary segments were identified: alarmed, concerned, cautious, and disengaged-dismissive. The groups exhibited distinctive personal characteristics and decreasing levels of policy support and involvement. For example, those in segment “Disengaged- Dismissive” were generally younger and physically and psychologically distant from the sea, showed less recognition of the problems, and exhibited infrequent related behaviors (e.g., consuming local seafood), with lower values of the sea. Such knowledge informs policymakers in targeting decisions. The two messaging conditions comprised varied impacts on different segments, suggesting that policymakers should select messaging conditions based on the target segment. Our study highlighted the importance of audience segmentation and targeted messaging in enabling policymakers to effectively reach the public sector.

**Supplementary Information:**

The online version contains supplementary material available at 10.1038/s41598-026-38402-0.

## Introduction

Coastal areas are home to over 500 million people and are rich in ecosystems, but they are under severe threat from various pressures, including overpopulation and global climate change^1^. Strengthening public understanding and gaining support for coastal management are critical coastal managerial components^2–4^. Coastal management targets the geographical interface between land and sea and the people living in and related to coastal areas, as people and environment are intertwined and inseparable. Although people benefit from marine ecosystem services in coastal areas^5,6^, socioeconomic activities (e.g., fishery and lifestyle) affect the environmental state^1^. Such relationships embedded in coastal areas are complex because of the interdependencies of several parts, characterized as a complex adaptive system producing emergent and system-wide patterns^7^. Therefore, as the social-ecological system (SES) framework argues, coastal management must address sustainability problems ascribed to deeply intertwined social and ecological systems^8^. Successful coastal management should be based on SES rather than on ecosystem management, which focuses purely on ecological systems^1,6^. Ignoring the influence of social systems on ecological systems can lead to unintended and undesirable SES states^9^. For example, coastal eutrophication has been caused by economic activities, and significant management efforts have been made to tackle this problem^10^. Therefore, enhancing public understanding of and support for coastal SES management is critical for effective coastal management.

At least five aspects of public understanding of and support for coastal SES management exist, namely accountability, aligned goals, backing, involvement, and human–nature connections. First, accountability that can be enhanced through promoting public understanding is a necessary condition for legitimate coastal management^4,10–12^. Second, management goals must be aligned with public preferences, else, an undesirable SES state and failure to gain support could ensue^4,13^. Although reflecting public desires is a critical factor^12,14^, the public must sometimes be convinced to align their management goals^4^. For example, as the Seto Inland Sea (SIS) in Japan is faced with oligotrophication, which causes fish catch decline and the bleaching of cultured nori (*Piropia yezoensis*), the Hyogo Prefectural Government implemented a nutrient management plan, including utilizing factory and sewage treatment plants (FSTP) to supply nutrients^4^. In this instance, negative public perceptions, such as pollution from FSTP, had to be addressed to gain public support for these necessary measures. Third, public understanding and support can facilitate the implementation of management measures when facing challenges from stakeholders with different interests^13,15^. For instance, public backing helped policymakers in Chesapeake Bay, USA, to implement more aggressive agricultural nonpoint-source controls^15^. Fourth, public understanding and support could accelerate the overall involvement in SES management, bringing about successful management^3,11,14^. The degree and method of involvement significantly affect SES^3,10^. The Hyogo Prefectural Government, which manages part of the SIS, requires public involvement by ordinance^16^. Fifth, involvement could further enhance public understanding and support, leading to greater involvement by strengthening human–nature connections^17^ Conservation studies claim that the disconnect between society and nature is the root cause of unsustainable SES^18–20^. Reconnecting people with nature can be a deep leverage point for the sustainability transformation of SES^21^. Experiences, such as involvement, can help people reconnect with nature^19,21,22^.

Capturing the heterogeneous characteristics of the public is crucial to effectively target them and promote their understanding of and support for environmental issues; however, earlier studies investigated the average effects of interventions only at the aggregate level^23–25^. For example, Uehara and Hidaka^4^ investigated public understanding of and support for SES coastal management at this level (i.e., the average of survey participants). Although stakeholder heterogeneity is well documented^26,27^, studies on the interest or disinterest of the public in SES issues and management including that of coastal areas are lacking^28^.

Audience segmentation is a promising approach to reveal the varying characteristics of the public and eliciting practical management implications for effective targeting of a heterogeneous public^25^. Audience segmentation is widely used in diverse research fields but is a relatively new approach in sustainability science^25,29^. The definition of audience segmentation is, “a process of identifying groups of people within a larger population who are homogeneous with regard to critical attributes (e.g., beliefs, behaviors, political ideology) most relevant to the objectives of a public engagement campaign”^29^. Various other methods, such as latent class modelling of a discrete choice experiment, are used in studies to determine public heterogeneity^30,31^ as well as empirical analyses to identify the factors influencing people^32^. However, audience segmentation is a more operational and efficient method because it intends to maximize the impact on people by targeting groups sharing similar antecedent qualities, such as knowledge and concerns, by delivering tailored messages^25,33^. By identifying segments and tailoring messages accordingly, target populations can be reached effectively. Uehara et al.^3^ revealed the socio-demographic heterogeneities of coastal residents, indicating the effectiveness of this approach for coastal SES management.

Our study aimed to reveal the heterogeneity of the public, their support for policy, attitudes, and involvement in coastal SES management through audience segmentation and targeted messaging to elicit practical management implications. Accordingly, three research questions were posed:

RQ1. How different are the sociodemographic characteristics of the public by audience segment concerning their opinions on coastal SES issues and management?

RQ2. How different are the levels of support for government-led coastal SES management measures according to audience segments and messaging conditions?

RQ3. How different are attitudes and involvement in coastal SES management according to audience segments and messaging conditions?

We applied audience segmentation to coastal SES management in the SIS of Japan, where the prefectural government implemented four primary nutrient supply measures for oligotrophication abatement. The effectiveness of two messages in targeting audience segments was assessed. Uehara et al.^4^ studied the same area at an average level, but did not investigate heterogeneity. We collected a new dataset from newly recruited participants for our study. Due to the paucity of studies using audience segmentation in sustainability science^25^, our study not only contributes to coastal SES management but also provides insights into the usefulness of this approach in sustainability science.

## Materials and methods

We employed a treatment–control design, with respondents sampled from the targeted public being randomly split into three groups: a control group without a message and two treatment groups with two different messaging conditions. This study was approved by the ethics committee of Ritsumeikan University (Kinugasa-Hito-2024-78). All methods were performed in accordance with the relevant guidelines and regulations stipulated by the ethics committee.

### Study area

**Fig. 1:**
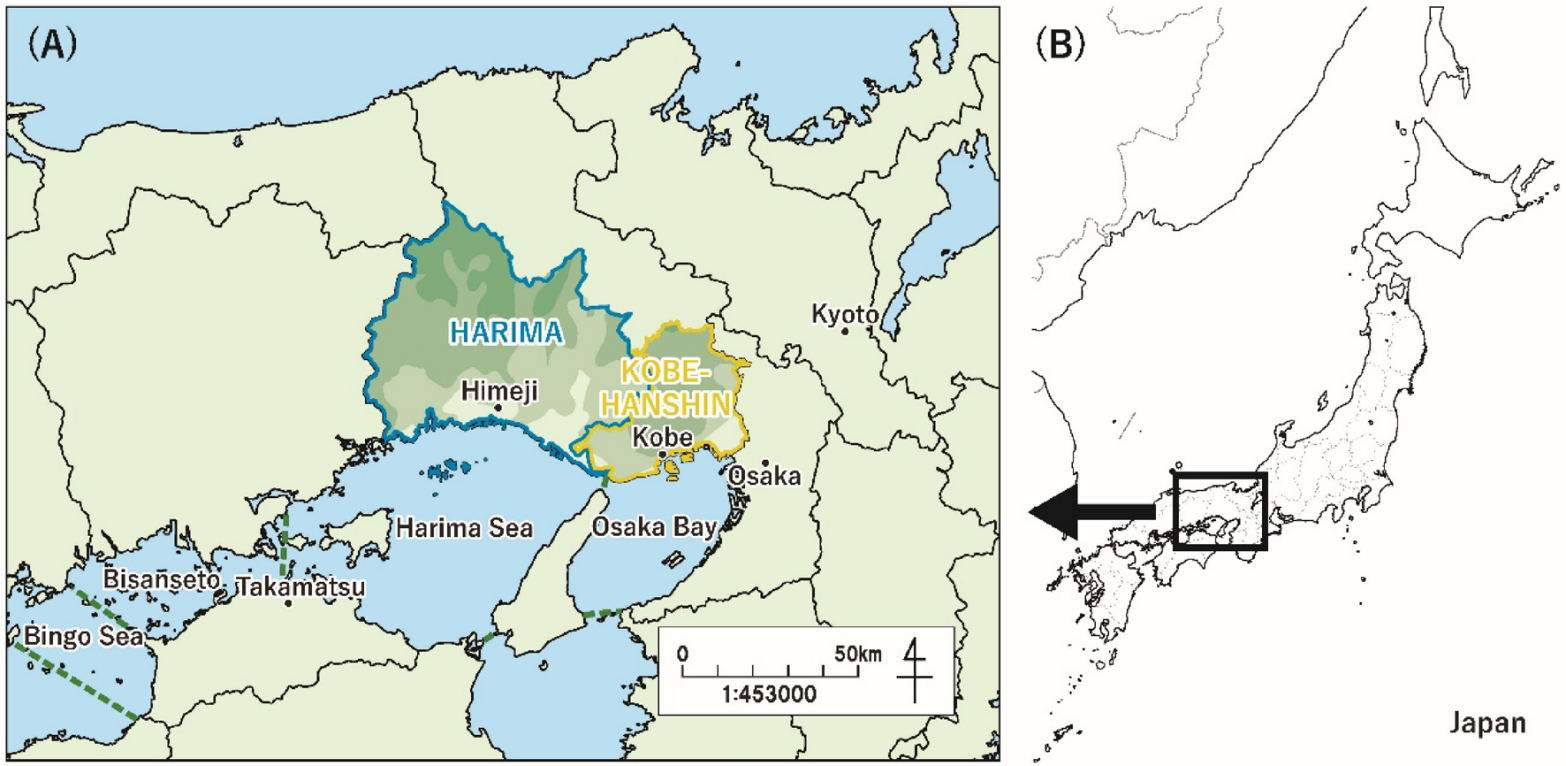
Location of the coastal SES in this study.

We investigated a coastal SES, which is managed by the Hyogo Prefectural Government. The area included part of the SIS (Harima Sea and Osaka Bay) and a part under the jurisdiction of the Hyogo Prefectural Government (Harima and Kobe-Hanshin regions), as defined by Uehara et al.^3^ (Fig. 1). The northern boundaries of the regions were located approximately along the drainage divide of the SIS.

Legislation on Special Measures Concerning Conservation of the Environment of the Seto Inland Sea of Japan was enacted in 1973 to deal with cultural eutrophication ascribed to human activity polluting the seawater. However, the long-term application of strict regulations led to oligotrophication, fish catch decline, and the bleaching of cultured nori (*Piropia yezoensis*)^34–36^. The stock of juvenile sand lances (*Ammodytes personatus*), a culturally important signature species of the SIS, has been extremely low for 9 years, and fishing was terminated earlier than that of the scheduled period to protect the remaining stock^37^.

Consequently, the Hyogo Prefectural Government, the entity responsible for coastal SES management and part of the SIS facing Hyogo Prefecture, implemented a nutrient supply plan to solve the oligotrophication issue^38^. The plan included four primary measures: (1) nutrient supply from factories and sewage treatment plants (FSTPs), (2) fertilization, (3) seabed plowing, and (4) drainage. Furthermore, an ordinance was enacted and issued requiring the public to contribute to revitalizing the richness and beauty of the SIS through their own life styles and community activities^16^. Public understanding of and support for nutrient supply from FSTPs are particularly important because of negative preconceptions about “polluting the coastal sea”^4^. Public understanding of and support for coastal SES management, including the four nutrient supply measures, were studied before, but only at the average level, and the heterogeneity of the public is not known^4^.

### Data collection

Participants were recruited from the target resident group (i.e., people living in the Harima or Kobe-Hanshin regions, shown in Fig. 1) registered at a survey company (Cross Marketing, Inc.), and the survey was conducted online between February 18 and 26, 2025. The sex distribution matched that of the population (52% female and 48% male), although the age distribution differed slightly because of survey panel availability (Table A1 in the Supplementary Material (SM1)). Participants were randomly assigned to either the control or one of the two treatment groups. Participants who failed the comprehension test were excluded from the survey. We recruited 600 valid participants from each group (1800 in total). We obtained informed consent from all the participants. All data are available as supplementary material (SM2) for replicability.

### Questionnaire design

The questionnaire comprised three components: (1) respondent characteristics, (2) support for the nutrient supply plan, and (3) attitudes toward and involvement in coastal SES management. All groups answered the same questions, except for the messaging conditions in the second component. The survey is provided as supplementary material (SM3).

The questions that were asked about respondent characteristics were selected based on two criteria: whether they explain differences across audience segments and are informative for eliciting management implications. In addition to basic attributes, including age, sex, and education, the distance of the respondents from the sea and their perceptions of the problems, behaviors, and values of nature were queried, as these factors could influence message effectiveness^2^. Distance from the sea includes both physical and psychological measures, with the physical measure being the time required to reach the sea from home. The psychological measure employed the Inclusion of Nature in Self (INS) scale, which measures the psychological dimension of human-nature relationships^17,39^. Human-nature relationships are positively associated with attitudes and behaviors toward a sustainable SES^19,40^. Perception relates to the decline in fish catch and oligotrophication (1. never heard of, … 5. know well for both questions). Behaviors include the frequency of visiting the sea (1. rarely, … 5. more than once a week), consuming local seafood (1. Yes, 2. No, 3. I don’t know), and participating in related events in Hyogo Prefecture (1. Yes, 2. No, 3. I don’t know). Values can address differences in understanding of and support for coastal SES management because they constitute the motivational basis of attitudes and behaviors^41,42^. Following the value pluralism approach to avoid eliciting biased management implications^43,44^, our questions are related to three value types: intrinsic, instrumental, and relational^45,46^. We adopted four, seven, and two items for the instrumental, relational, and intrinsic values of the SIS, respectively (Table 1), which were evaluated in earlier studies^3,40^. We used a 5-point Likert scale ranging from “1. Strongly disagree” to “5. Strongly agree.”

**Table 1:** Value statements of instrumental, relational, and intrinsic values.

Value class	Statement
Instrumental	The SIS is important to me, because it provides food, such as seafood
	The marine ecosystem of the SIS is important to me as a means for preventing and mitigating disasters, such as floods, tsunamis, and typhoons
	The marine ecosystem of the SIS is important to me because it purifies the seawater
	The SIS is important to me, because it maintains the food chain (the relationship between animals and plans of eating and being eaten)
Relational	The SIS is an important location for me
	The SIS is an important location for local residents
	I can connect with others through my relationship to the SIS
	Caring for the SIS leads to caring for the people of the present and future
	We have a moral responsibility to protect the SIS and its creatures
	Protecting the SIS fills me with a sense of contentment and enables me to lead a good life
	Maintaining the SIS in good condition is the right thing to do
Intrinsic	Every living organism in the Seto Inland Sea has the right to live
	The Seto Inland Sea should be protected for nature itself, regardless of whether it is for us or not

Before querying the support of the respondents for the nutrient supply plan, we asked their opinion on the current state of the sea (oligotrophication) and the need for active intervention. We used this question for audience segmentation regarding their understanding and support. We adopted the segmentation proposed and tested in the cases of global warming and wildlife conservation^25,29,47,48^. The segmentation has two theoretical dimensions: attitudinal valence and issue involvement. Attitudinal valence is a linear relation of concern across six categories (from more acceptance in the Alarmed to less acceptance in the Doubtful), and issue involvement is a J-shaped curvilinear relation on outcomes related to engagement in the topic, which is a downward relation but slightly increasing among the Dismissive^47,49^. The segmentation is also grounded in the diffusion of innovation theory, which explains how new ideas, norms, and behaviors are adopted through distinct segments^25,50^.

In this study, a single-item self-categorization measure comprising six statements was adopted as an alternative to the multiple-item screener that utilizes multiple items for self-categorization^49^, as listed below. Respondents were asked to choose the statement closest to their opinions.

1. *Alarmed:* I am very concerned about the oligotrophication of the SIS and I believe that governments and individuals should take immediate action.
2. *Concerned:* I am concerned about the oligotrophication of the SIS and believe that action must be taken although time to determine an appropriate response still exists.
3. *Cautious:* I think the SIS is becoming oligotrophic, although I am not sure. We must carefully determine the timing of and mechanism for interventions.
4. *Disengaged:* I have not thought much about the SIS becoming oligotrophic.
5. *Doubtful:* I do not think the SIS has become oligotrophic, although I am not certain. I am more concerned about an overreaction to the assumed oligotrophication.
6. *Dismissive:* I do not think neither oligotrophication of the SIS is occurring nor that humans have caused it. Therefore, I am neither inclined to take action to address oligotrophication nor do I support such action.

Shorter measures of psychological phenomena were developed to reduce the burden on the respondents^51,52^. These measures were applied in environmental issues, such as climate change^25,49^ and wildlife conservation^25^. Swim et al.^49^ developed a single-item self-categorization measure from the original 36-item screener developed in the Six Americas project to segment the climate change opinions among the American public^29,47^ and confirmed the reliability, construct validity, and predictive validity, and the results support attitudinal valence and issue involvement. Naito et al.^25^ also validated its validity in their study on wildlife conservation. The six statements in our study were developed by tailoring the statements used in these earlier studies to our objective^25,49^. We consulted policymakers who were knowledgeable of the coastal SES management in developing the following statements by following one of the two item selection strategies^53^. Furthermore, before developing them, we answered three basic guiding questions for short scale construction: the construct to be measured, the main purpose of the short scale and the targeted population of the short scale^52^. Our measure targets residents in the study area to segment them based on their understanding and support for the nutrient supply, aiming to elicit implications for gaining more support and involvement in coastal SES management.

Because these statements include policy intervention support, we could expect different segments to report varying levels of support for government-led coastal SES management and involvement in coastal SES management. Our intention, however, is not only to investigate such a potentially monotonic relationship but also to examine how each segment perceives specific government-led coastal SES management measures and involvement in coastal SES management. For example, some measures (e.g., FSTPs) may be controversial even among people who are concerned about the oligotrophication of the SIS^4^. Furthermore, this study also aims to reveal the socio-demographic characteristics of each segment (RQ1).

Support for the four nutrient supply measures (i.e., nutrient supply from FSTP, fertilization, seabed plowing, and drainage) was measured using a 6-point Likert scale ranging from “1. Completely not supportive” to “6. Completely supportive.” As the respondents could be unfamiliar with these measures, pictures with additional explanations where required were provided (see SM3). The pictures were used by the Hyogo Prefectural Government to explain these measures. Furthermore, the impressions of the respondents of the measures were qualitatively analyzed using an open-ended question.

To measure the attitudes toward and involvement of the respondents in coastal SES management, we asked two types of questions: the general desire to work for coastal SES^17^ and specific attitudes and behaviors. A gap could exist between the general agreement on this issue and agreement on the particular aspects. For the latter, we adopted the pro-SES behavior scale developed and validated by Uehara et al.^3^. The former included one question (“Do you want to contribute to making the Seto Inland Sea a richer place?”), and the latter included six questions [desirable coastal SES state, consuming local seafood, proper plastic waste management, conservation of mudflats and seagrass beds, forest conservation, cleaning beaches, and rivers (see SM3 for details)]. A 5-point Likert scale ranging from “1. Strongly disagree” to “5. Strongly agree” was used in the measurements.

We asked respondents about their intention to join the “Hyogo Prefectural Citizens’ Council for the Development of an Abundant Sea,” managed by the Hyogo Prefectural Government. The survey company that administered the online survey tracked whether respondents clicked the URL to register for the council. The council provided information about related events and connected members. Registration was free. Click-through rates can be viewed as an indicator of actual involvement behavior, as opposed to intentions^25^.

### Messaging conditions

We designed three messaging conditions to investigate the influence on the respondents in each audience segment, which are the “no information” control, factual message, and moral messaging conditions^2,54^. Several studies provided a baseline message as a control condition^2,25^ but we chose the “no information” condition to investigate respondent opinions without providing any additional information^54^. Our strategies were inspired by Dean et al.^2^, who studied the effectiveness of factual and moral arguments in gaining community support for coastal management, as well as by Naito et al.^25^, who examined the impact of messaging conditions on the attitudes and behaviors of segmented respondents toward exotic pets.

**Fig. 2:**
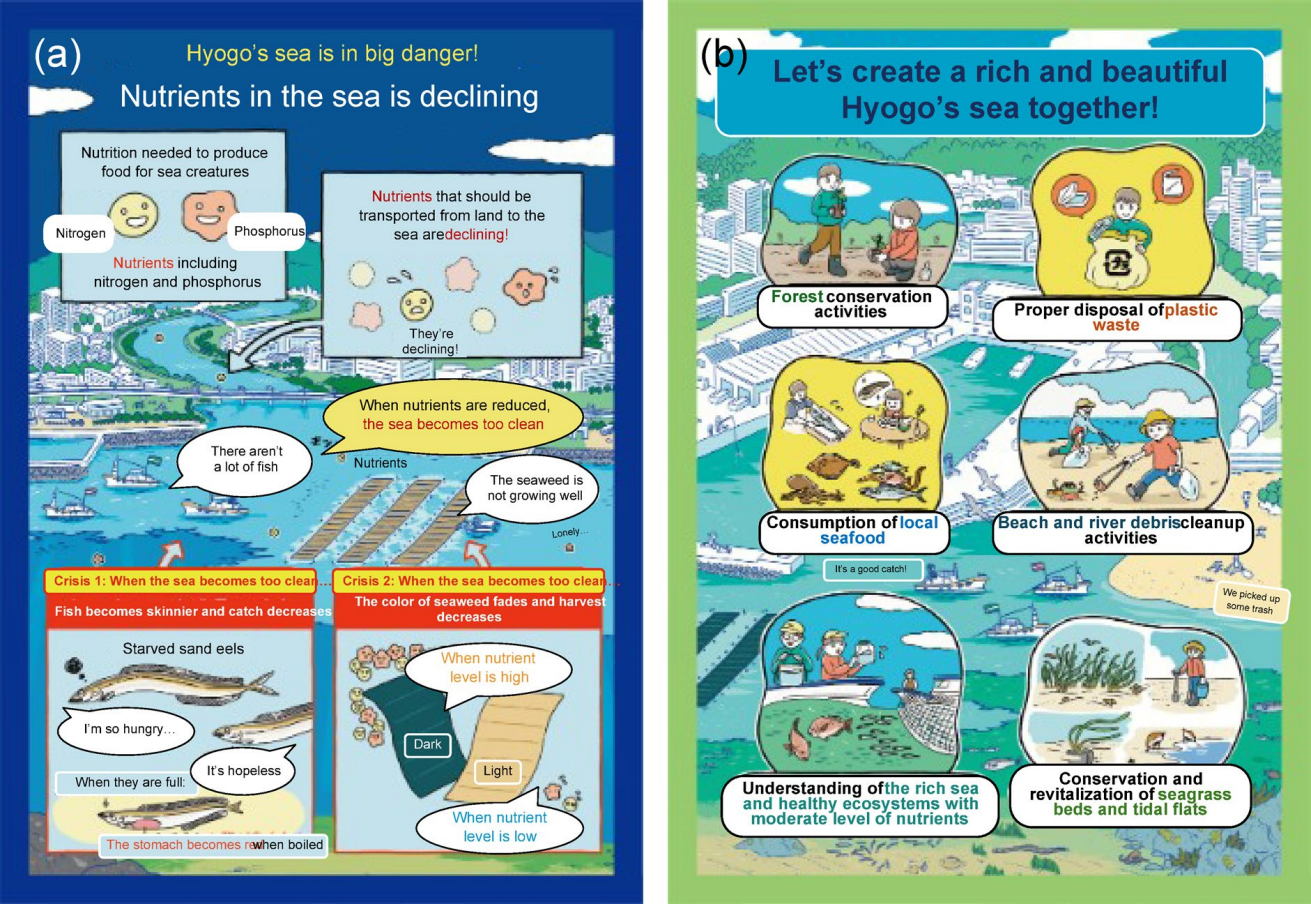
Message conditions. (**a**) M1: negative consequences of oligotrophication (left), and (**b**) M2: collective public involvement in coastal SES management to realize the bounty and beauty of the sea (right). “Hyogo’s sea” is the part of the SIS under jurisdiction of the Hyogo Prefectural Government.

Our first message (M1) emphasized the negative consequences of oligotrophication (Fig. 2a), a type of factual argument^2^. Two negative impacts of the lack of nutrients on fish and seaweed were emphasized. The second message (M2) encouraged the collective involvement of the public in coastal SES management to realize the bounty and beauty of the sea (Fig. 2b), a type of moral argument^2^. Six types of activities (e.g., Forest conservation and consuming local seafood) were promoted. Collective action can be motivated by moral obligation^55,56^. To highlight the differences, we used different color combinations and background coastal zones. For example, red was used in M1 to highlight the problems. We consulted the Hyogo Prefectural Government to assess the validity of the content. M2 was consistent with that of the queries from the Hyogo Prefectural Government addressed to the public, and queries concerning pro-SES behavior developed by Uehara et al.^3^. The comprehension of the respondents regarding the message was assessed (see SM3), and those who chose an incorrect answer twice in a row were excluded from the survey.

### Data analysis

The collected data were analyzed by audience segment to determine the similarities and differences between the segments. The Scheffé test for post-hoc comparisons in analysis of variance was conducted to investigate the differences in the mean scores of the characteristics of the respondents by segment^48^. The test is suitable for exploratory data analysis where the theoretical foundations or earlier studies on differences between groups are not sufficient^57^. A comparison was conducted between the mean values of the support by the respondents for a nutrient supply plan, attitudes, and involvement in coastal SES management and the 95% confidence intervals for the population mean. ANOVA tests with effect sizes based on partial eta squared (${\eta }_{p}^{2}$) were conducted to assess the main effects of messaging conditions and segmentations, and the interaction effects between them. ${\eta }_{p}^{2}$ can be interpreted as small if < 0.06, medium if 0.06 to < 0.14 and large otherwise^58^. Data analysis was conducted using StataBE-18 (64-bit) (https://www.stata.com). We present verbatim quotations from open-ended questions as exemplars to complement the quantitative analyses^59^. Verbatim quotations were selected by the lead author and validated by co-authors.

## Results

### Respondent characteristics

The characteristics of the respondents by segment are summarized in Table 2. The Cronbach’s alphas for the instrumental, relational, and intrinsic values were 0.927, 0.922, and 0.879, respectively, indicating internal consistency as a scale^60^. Figures in the same row sharing superscripts differed at the 5% level in the post-hoc test (Scheffe’s test). For example, the male ratio in “2. Concerned” was significantly higher than that in “4–6. Disengaged–Dismissive.” All characteristics showed some differences between the audience segments. All the characteristics, except male ratio and higher education, showed a downward relationship.

**Table 2:** Summary of respondents by segment (N = 1800).

	1. Alarmed	2. Concerned	3. Cautious	4–6. Disengaged–dismissive
*N*	347	489	454	510
Demographic				
Male (in %)	49.9%	53.8%^a^	46.9%	42.2%^a^
Age	59.7^a^	55.5^a,b^	54.1^a,c^	45.4^a,b,c^
Higher education (in %) (undergraduate and graduate levels)	59.4%	62.2%^a^	62.8%^b^	51.6%^a,b^
Distance				
Physical (min)	51.3^a^	53.5^b^	57.5	63.8^a,b^
INS scale (1. weakest to 7. strongest)	2.40^a^	2.21^b^	2.00^a,c^	1.55^a,b,c^
Perception of the problem				
Fishery (1. never heard of, … 5. know well)	2.65^a^	2.28^a,b^	1.86^a,b,c^	1.48^a,b,c^
Oligotrophication (1. never heard of, … 5. know well)	2.67^a^	2.27^a,b^	1.76^a,b,c^	1.42^a,b,c^
Behavior				
Visit (1. rarely, … 5. more than once a week)	2.44^a^	2.35^b^	2.17^a,c^	1.61^a.b,c^
Consuming local seafood (in %)	26.2%^a^	26.8%^b^	19.8%^c^	6.1%^a,b,c^
Event (in %)	3.2%^a^	2.5%^b^	0.9%	0.2%^a,b^
Value (1. Totally disagree,…, 5. Totally agree)				
Instrumental	3.99^a^	3.80^a,b^	3.61^a,b,c^	3.02^a,b,c^
Relational	4.02^a^	3.86^a,b^	3.63^a,b,c^	3.09^a,b,c^
Intrinsic	4.35^a^	4.19^a,b^	3.95^a,b,c^	3.53^a,b,c^

Figures represent means, unless indicated otherwise. Figures in the same row sharing superscripts differed at the 5% level in the post-hoc test (Scheffe’s test).

**Fig. 3:**
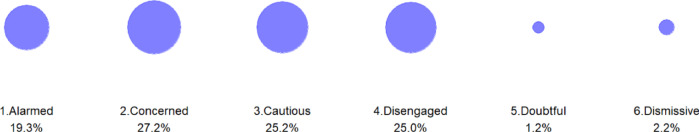
Audience segment composition (N = 1800).

Most respondents expressed affirmative perceptions on mitigation of the SIS issues, with nearly three-quarters (71.7%) choosing audience segments “1. Alarmed,” “2. Concerned,” or “3. Cautious.” However, 25% were indifferent (“4. Disengaged”) and 3.4% were suspicious (“5. Doubtful”), or negative (“6. Dismissive”), as shown in Fig. 3. The breakdown of segments by group (Table A2 in the Supplementary Material (SM1)) showed that the percentage of “6. Dismissive” was prominently lower in M1 (0.5%) and M2 (0%) than that in the control group (6.0%). Due to the questionnaire design, those in this segment may have been underrepresented in groups M1 and M2. Although the question about the messages was a comprehension test and not about the level of agreement, some respondents who belonged in this segment probably did not answer correctly and were eliminated from the survey. Therefore, the number of respondents who answered “6. Dismissive” in groups M1 and M2 may have been underestimated.

The number of respondents in segments 5 and 6 was small, even for that in the control group, and it is possible to analyze them as segments with a less positive attitude toward problem solving^25^. We analyzed segments 4 through 6 collectively as “4–6. Disengaged–Dismissive” hereafter. Although segment “4. Disengaged” and segments “5. Doubtful” and “6. Dismissive” could have different attitudes, they were treated together here as a segment with a non-positive orientation toward the proposed measures. However, combined, negative segments “5. Doubtful” and “6. Dismissive” was relatively small (3.4%).

### Support for government-led coastal SES management measures

Table 3 shows the summary of respondents’ support for nutrient supply measures, attitudes and involvement in coastal SES management, and intention and behavior of joining the Hyogo Prefectural Citizens’ Council for the Development of an Abundant Sea. As for the support for nutrient supply measures, the significant main effects of messing conditions and segments were observed at 5% significance level in all measures, except for that of messaging conditions on “FSTP.” The interaction effect was observed only for “Drainage.” All size effects were either small (< 0.06) or medium (0.06 to < 0.14). As for the attitudes and involvement in coastal SES management, the significant main effects of messing conditions and segments were observed at 5% significance level in all attitudes and involvement except for those of messaging conditions on “Conservation of seaweed beds and tidal flats” to “Cleanup beaches and rivers.” The interaction effect was observed only for “General intention to contribute.” Effect sizes for segments were large except for “General intention to contribute (${\eta }_{p}^{2}$= 0.139, medium),” “Consuming local seafood (${\eta }_{p}^{2}$= 0.130, medium),” and “Plastic waste disposal (${\eta }_{p}^{2}$= 0.079, medium).” The main effects of messaging conditions and segments for “intention to join” were statistically significant at 5% level, but not for the interaction effect. The size effect of segments was large. No effects were observed regarding the click action.

**Table 3:** Summary of respondents’ support, attitudes, involvement, intention, and behavior by group and segment with ANOVA and effect sizes.

	1. Alarmed	2. Concerned	3. Cautious	4–6. Disengaged–dismissive	ANOVA and Effect size (partial η^2^)
	Mean ± SD	Mean ± SD	Mean ± SD	Mean ± SD	
Support for nutrient supply measures					
All measures					
Control	5.08 ± 0.93	4.77 ± 0.89	4.38 ± 0.8	4.1 ± 0.76	Group: *F*(2, 1800) = 9.15, *p* < 0.001, ${\eta }_{p}^{2}$ = 0.01
M1	5.2 ± 1.01	4.87 ± 0.89	4.73 ± 0.74	4.4 ± 0.78	Segment: *F*(3, 1800) = 80.36, *p* < 0.001, ${\eta }_{p}^{2}$ = 0.119
M2	5.18 ± 1	4.81 ± 0.9	4.4 ± 0.82	4.37 ± 0.76	Group × Segment: *F*(6, 1800) = 1.79, *p* = 0.097, ${\eta }_{p}^{2}$ = 0.006
1. FSTP					
Control	4.69 ± 1.17	4.26 ± 1.26	4.07 ± 1.09	3.96 ± 0.94	Group: *F*(2, 1800) = 0.02, *p* = 0.978, ${\eta }_{p}^{2}$ = 0.00
M1	4.52 ± 1.68	4.27 ± 1.3	4.21 ± 1.12	3.96 ± 1.1	Segment: *F*(3, 1800) = 18.94, *p* < 0.001, ${\eta }_{p}^{2}$ = 0.031
M2	4.61 ± 1.57	4.33 ± 1.21	3.99 ± 1.2	4.09 ± 1.06	Group × Segment: *F*(6, 1800) = 0.86, *p* = 0.523, ${\eta }_{p}^{2}$ = 0.003
2. Fertilization					
Control	4.98 ± 1.26	4.62 ± 1.12	4.33 ± 0.94	4.13 ± 0.89	Group: *F*(2, 1800) = 3.85, *p* = 0.021, ${\eta }_{p}^{2}$ = 0.004
M1	5.03 ± 1.29	4.8 ± 1.17	4.58 ± 0.98	4.36 ± 0.94	Segment: *F*(3, 1800) = 39.34, *p* < 0.001, ${\eta }_{p}^{2}$ = 0.062
M2	5.07 ± 1.2	4.69 ± 1.15	4.33 ± 1.1	4.32 ± 0.99	Group × Segment: *F*(6, 1800) = 0.57, *p* = 0.753, ${\eta }_{p}^{2}$ = 0.002
3. Seabed plowing					
Control	5.14 ± 1.13	4.93 ± 1.14	4.49 ± 1.06	4.16 ± 0.91	Group: *F*(2, 1800) = 16.11, *p* < 0.001, ${\eta }_{p}^{2}$ = 0.018
M1	5.43 ± 1.21	5.14 ± 1.08	4.98 ± 0.89	4.59 ± 1.02	Segment: *F*(3, 1800) = 58.74, *p* < 0.001, ${\eta }_{p}^{2}$ = 0.09
M2	5.35 ± 1.24	5.02 ± 1.06	4.56 ± 1.11	4.48 ± 0.89	Group × Segment: *F*(6, 1800) = 1.23, *p* = 0.2853, ${\eta }_{p}^{2}$ = 0.004
4. Drainage					
Control	5.51 ± 1.24	5.26 ± 1.19	4.64 ± 1.16	4.15 ± 0.94	Group: *F*(2, 1800) = 13.85, *p* < 0.001, ${\eta }_{p}^{2}$ = 0.015
M1	5.82 ± 1.05	5.26 ± 1.14	5.13 ± 1.04	4.68 ± 1.01	Segment: *F*(3, 1800) = 95.42, *p* < 0.001, ${\eta }_{p}^{2}$ = 0.138
M2	5.68 ± 1.08	5.21 ± 1.11	4.7 ± 1.01	4.61 ± 1.01	Group × Segment: *F*(6, 1800) = 3.2, *p* = 0.004, ${\eta }_{p}^{2}$ = 0.011
Attitudes and involvement in coastal SES management					
General intention to contribute					
Control	3.81 ± 0.87	3.58 ± 0.78	3.51 ± 0.66	3.16 ± 0.8	Group: *F*(2, 1800) = 6.38, *p* = 0.002, ${\eta }_{p}^{2}$ = 0.007
M1	4.07 ± 0.71	3.66 ± 0.78	3.64 ± 0.61	3.16 ± 0.78	Segment: *F*(3, 1800) = 96.3, *p* < 0.001, ${\eta }_{p}^{2}$ = 0.139
M2	4.06 ± 0.63	3.88 ± 0.7	3.57 ± 0.64	3.13 ± 0.77	Group × Segment: *F*(6, 1800) = 2.6, *p* = 0.017, ${\eta }_{p}^{2}$ = 0.009
All intentions and behaviors					
Control	4.05 ± 0.67	3.83 ± 0.61	3.64 ± 0.58	3.23 ± 0.63	Group: *F*(2, 1800) = 11.8, *p* < 0.001, ${\eta }_{p}^{2}$ = 0.013
M1	4.2 ± 0.54	3.92 ± 0.55	3.77 ± 0.46	3.33 ± 0.64	Segment: *F*(3, 1800) = 167.16, *p* < 0.001, ${\eta }_{p}^{2}$ = 0.219
M2	4.19 ± 0.52	4.07 ± 0.51	3.72 ± 0.46	3.42 ± 0.61	Group × Segment: *F*(6, 1800) = 1.12, *p* = 0.350, ${\eta }_{p}^{2}$ = 0.004
1. Desired state					
Control	4.24 ± 0.7	3.95 ± 0.71	3.77 ± 0.71	3.33 ± 0.74	Group: *F*(2, 1800) = 15.41, *p* < 0.001, ${\eta }_{p}^{2}$ = 0.017
M1	4.32 ± 0.63	4.11 ± 0.61	4.03 ± 0.5	3.56 ± 0.76	Segment: *F*(3, 1800) = 125.98, *p* < 0.001, ${\eta }_{p}^{2}$ = 0.174
M2	4.35 ± 0.6	4.19 ± 0.55	3.9 ± 0.51	3.57 ± 0.64	Group × Segment: *F*(6, 1800) = 1.22, *p* = 0.295, ${\eta }_{p}^{2}$ = 0.004
2. Consuming local seafood					
Control	4.07 ± 0.81	3.8 ± 0.75	3.65 ± 0.75	3.21 ± 0.77	Group: *F*(2, 1800) = 9.11, *p* < 0.001, ${\eta }_{p}^{2}$ = 0.010
M1	4.15 ± 0.75	3.85 ± 0.68	3.83 ± 0.71	3.34 ± 0.78	Segment: *F*(3, 1800) = 89.1, *p* < 0.001, ${\eta }_{p}^{2}$ = 0.130
M2	4.19 ± 0.74	4.07 ± 0.64	3.73 ± 0.68	3.48 ± 0.83	Group × Segment: *F*(6, 1800) = 1.56, *p* = 0.154, ${\eta }_{p}^{2}$ = 0.005
3. Plastic waste disposal					
Control	4.42 ± 0.82	4.17 ± 0.88	4.03 ± 0.91	3.59 ± 0.9	Group: *F*(2, 1800) = 23.43, *p* < 0.001, ${\eta }_{p}^{2}$ = 0.026
M1	4.57 ± 0.6	4.36 ± 0.71	4.26 ± 0.69	3.96 ± 0.93	Segment: *F*(3, 1800) = 51.38, *p* < 0.001, ${\eta }_{p}^{2}$ = 0.079
M2	4.65 ± 0.59	4.45 ± 0.64	4.24 ± 0.81	4.11 ± 0.9	Group × Segment: *F*(6, 1800) = 1.45, *p* = 0.193, ${\eta }_{p}^{2}$ = 0.005
4. Conservation of seaweed beds and tidal flats					
Control	3.87 ± 0.84	3.68 ± 0.72	3.44 ± 0.65	3.07 ± 0.7	Group: *F*(2, 1800) = 1.65, *p* = 0.193, ${\eta }_{p}^{2}$ = 0.002
M1	4.03 ± 0.69	3.7 ± 0.74	3.52 ± 0.68	3.01 ± 0.84	Segment: *F*(3, 1800) = 129.35, *p* < 0.001, ${\eta }_{p}^{2}$ = 0.178
M2	3.96 ± 0.73	3.88 ± 0.68	3.44 ± 0.62	3.08 ± 0.76	Group × Segment: *F*(6, 1800) = 1.69, *p* = 0.121, ${\eta }_{p}^{2}$ = 0.006
5. Conservation of forest					
Control	3.94 ± 0.79	3.71 ± 0.78	3.5 ± 0.66	3.09 ± 0.77	Group: *F*(2, 1800) = 2.81, *p* = 0.061, ${\eta }_{p}^{2}$ = 0.003
M1	4.07 ± 0.69	3.77 ± 0.75	3.53 ± 0.69	3.04 ± 0.89	Segment: *F*(3, 1800) = 125.81, *p* < 0.001, ${\eta }_{p}^{2}$ = 0.174
M2	4.03 ± 0.72	3.92 ± 0.65	3.56 ± 0.65	3.14 ± 0.82	Group × Segment: *F*(6, 1800) = 0.84, *p* = 0.54, ${\eta }_{p}^{2}$ = 0.003
6. Cleanup beaches and rivers					
Control	3.78 ± 0.95	3.69 ± 0.8	3.45 ± 0.66	3.09 ± 0.74	Group: *F*(2, 1800) = 2.34, *p* = 0.097, ${\eta }_{p}^{2}$ = 0.003
M1	4.03 ± 0.69	3.73 ± 0.78	3.45 ± 0.75	3.04 ± 0.9	Segment: *F*(3, 1800) = 107.7, *p* < 0.001, ${\eta }_{p}^{2}$ = 0.174
M2	3.93 ± 0.68	3.9 ± 0.67	3.45 ± 0.65	3.11 ± 0.8	Group × Segment: *F*(6, 1800) = 1.63, *p* = 0.134, ${\eta }_{p}^{2}$ = 0.003
Intention to participate in the “Hyogo Prefectural Citizens’ Council for the Development of an Abundant Sea,” and the click-through rate on the URL of the website by segment and messaging condition					
Intention to join					
Control	3.99 ± 1.46	4.41 ± 1	4.54 ± 1.07	5.27 ± 1.23	Group: *F*(2, 1800) = 4.81, *p* = 0.008, ${\eta }_{p}^{2}$ = 0.005
M1	3.69 ± 1.37	4.01 ± 1.13	4.54 ± 1.02	5.19 ± 1.07	Segment: *F*(3, 1800) = 117.46, *p* < 0.001, ${\eta }_{p}^{2}$ = 0.165
M2	3.99 ± 1.14	4.11 ± 1.07	4.58 ± 1.08	5.24 ± 1.06	Group × Segment: *F*(6, 1800) = 1.31, *p* = 0.249, ${\eta }_{p}^{2}$ = 0.004
Click					
Control	0.62 ± 0.49	0.6 ± 0.49	0.63 ± 0.48	0.59 ± 0.49	Group: *F*(2, 1800) = 0.06, *p* = 0.945, ${\eta }_{p}^{2}$ = 0
M1	0.6 ± 0.49	0.57 ± 0.5	0.6 ± 0.49	0.63 ± 0.48	Segment: *F*(3, 1800) = 0.45, *p* = 0.720, ${\eta }_{p}^{2}$ = 0.001
M2	0.67 ± 0.47	0.61 ± 0.49	0.56 ± 0.5	0.61 ± 0.49	Group × Segment: *F*(6, 1800) = 0.72, *p* = 0.630, ${\eta }_{p}^{2}$ = 0.002

**Fig. 4:**
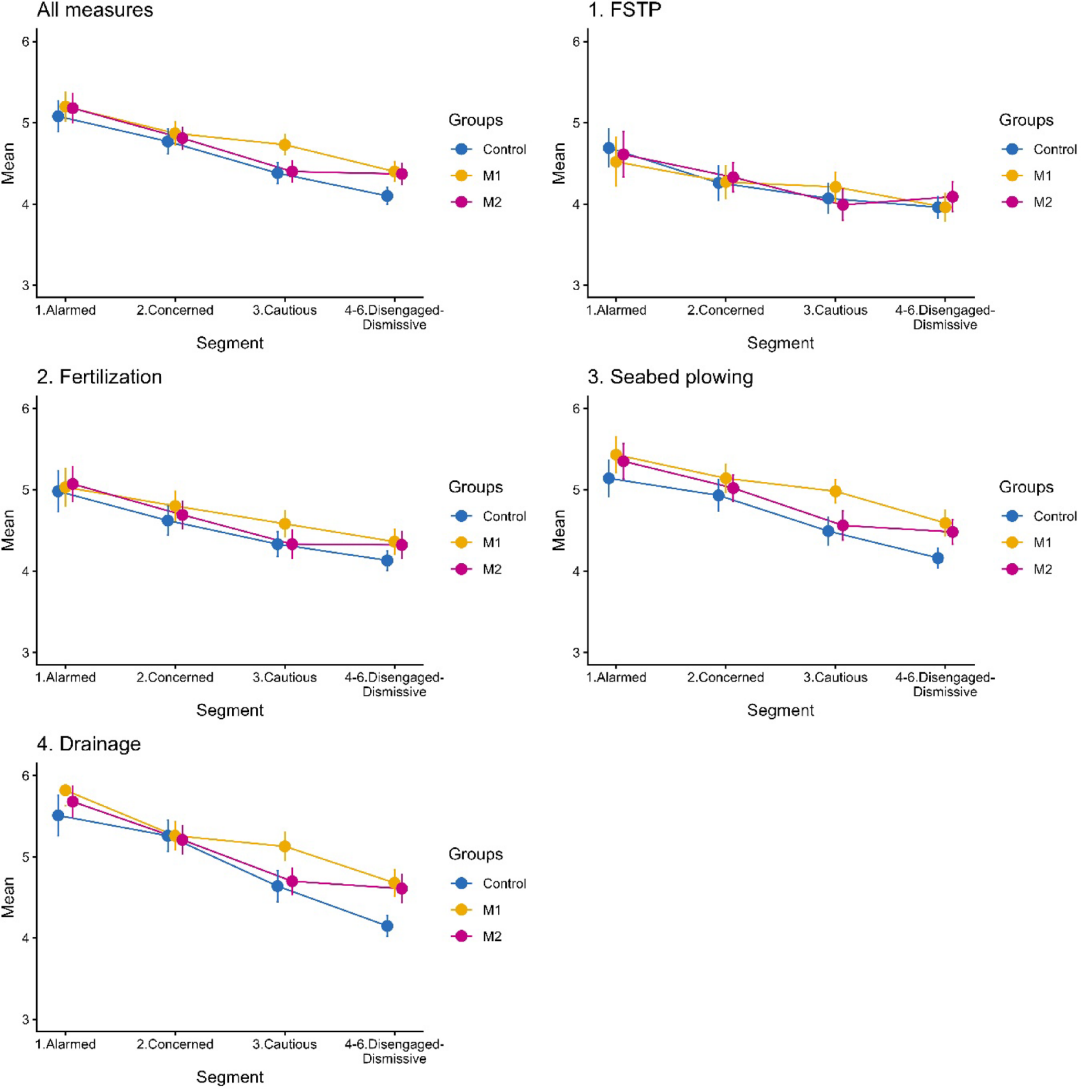
Support for nutrient supply measures by segment and messaging condition. Group 1: Control group, Group 2: M1 (negative consequences of oligotrophication), Group 3: M2 (collective public involvement). Error bars indicate 95% confidence intervals.

Figure 4 shows public support for the government-led coastal SES management measures (i.e., four nutrient supply measures) in the mean values of a 6-point Likert scale of “1. Completely not supportive” to “6. Completely supportive,” with 95% confidence intervals.

Regarding the control group without messages (blue line), for the mean of “All measures,” although the confidence intervals for segments “1. Alarmed” and “2. Concerned” overlapped, those for Segments “2. Concerned”; “3. Cautious” and “4–6. Disengaged–Dismissive” did not, indicating that the more positive the attitude toward solving the problem, the higher the level of support. The same trend was generally observed for each measure, with a particularly high level of support for “Drainage” among segment “1. Alarmed,” and a significant difference from Segment “4–6. Disengaged–Dismissive.” Overall, support for FSTP tended to be lower than that for the other measures in all segments. However, no statistically significant differences were observed for Segment “4–6. Disengaged–Dismissive” between the measures.

Regarding the impact of the messaging conditions [i.e., M1 (yellow line) or M2 (red line)] on the control group (blue line), no significant impact was observed on segments 1 and 2. However, Segments 3 and 4 showed different impacts. Regarding messaging M1 (Negative consequences of oligotrophication), the support for “Seabed plowing” and “Drainage” in segments “3. Cautious” and “4–6. Disengaged–Dismissive” was higher than that in the corresponding segments of the control group. Furthermore, support was as high as the next segment in the control group (e.g., the support for “Seabed plowing” in Segment “3. Cautious” was as high as that in Segment “2. Concerned” in the control group). Regarding messaging M2 (Collective public involvement), the support for “Seabed plowing” and “Drainage” in Segment “4–6. Disengaged–Dismissive” was higher than that in the corresponding segments of the control group. Similar to that in M1, support was as high as the next segment in the control group (e.g., Segment 4 in M2 for “Seabed plowing” and that of Segment 3 in the control group). No significant effect of the messaging conditions was observed for measures “FSTP” and “Fertilization.”

### Attitudes and involvement of respondents in coastal SES management

**Fig. 5:**
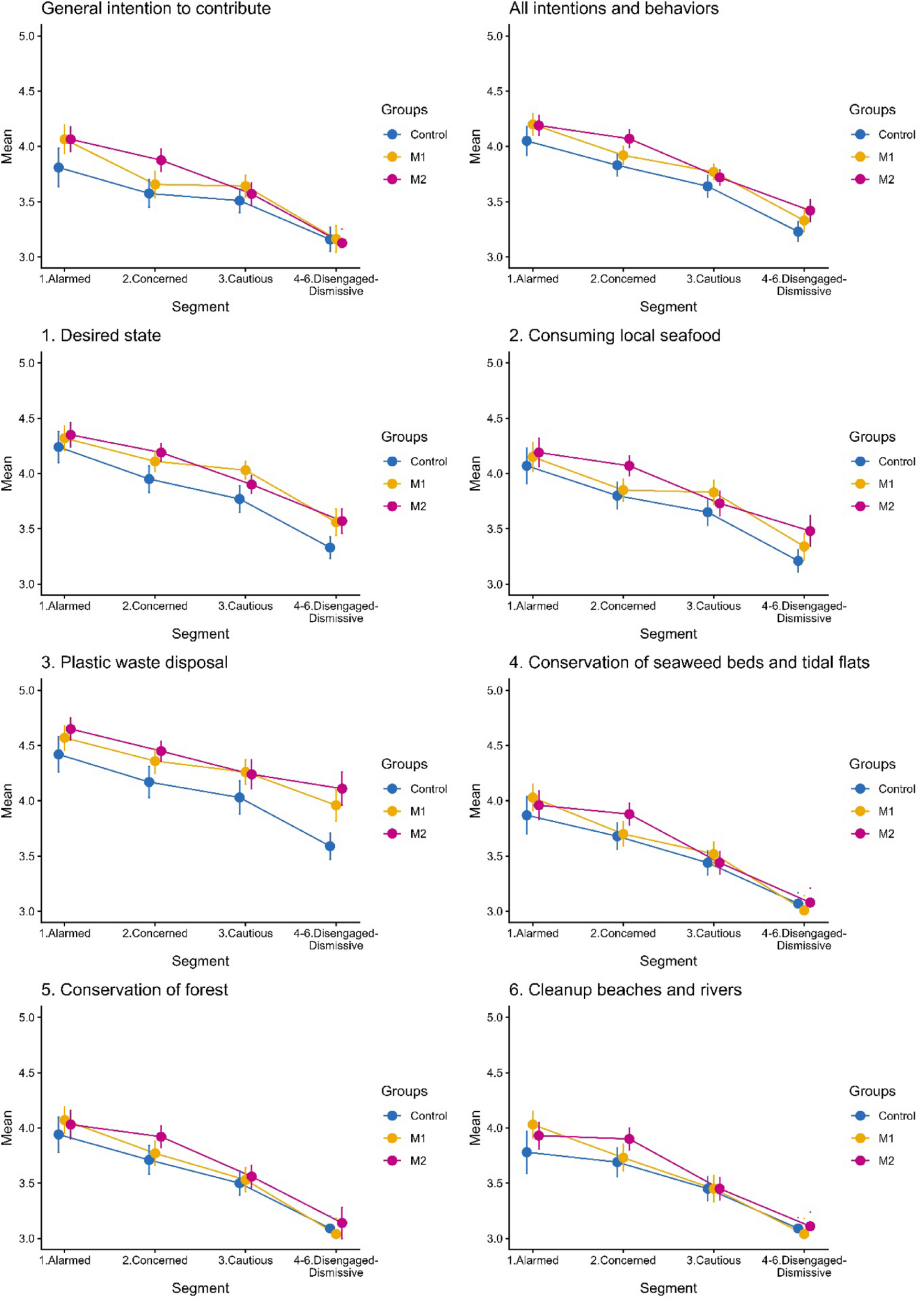
Attitudes and involvement of the respondents in coastal SES management by segment and messaging condition. Group 1: Control group, Group 2: M1 (negative consequences of oligotrophication), Group 3: M2 (collective public involvement). Error bars indicate 95% confidence intervals.

Figure 5 presents the attitudes and involvement of respondents in coastal SES management by message condition and segment, expressed as the mean values of a 5-point Likert scale ranging from “1. Completely disagree” to “5. Completely agree,” with 95% confidence intervals. Overall, the agreement levels differed monotonically from least in Segment “4–6. Disengaged–Dismissive” to most in Segment “1. Alarmed.” Regarding individual attitudes and actions, the respondents generally tended to agree highly on “Desired state” and “Plastic waste disposal,” whereas agreement on conservation activities such as “Conservation of seaweed bed and tidal flat,” “Conservation of forest,” and “Cleanup beaches and rivers” was generally low, with “Consuming local seafood” falling in between.

Regarding the impact of messaging M1 (Negative consequences of oligotrophication, yellow lines), the agreement levels on “Desired state” were higher in Segments “3. Cautious” and “4–6. Disengaged–Dismissive” than those of the control group. That of Segment “4–6. Disengaged–Dismissive” for “Plastic waste disposal” was also higher than the control group. The agreement level of Segment 3 in M1 was as high as that of Segment 1 in the control group for “Desired state,” and the agreement level of Segment 4 in M1 was as high as that of Segment 2 in the control group for “Plastic waste disposal.”

Regarding M2 (Collective public involvement, red lines), compared with the control group, higher agreement levels were observed for “Desired state” among Segments “2. Concerned” and “4–6. Disengaged–Dismissive”; for “Consuming local seafood” among Segments “2. Concerned” and “4–6. Disengaged–Dismissive”; and for “Plastic waste disposal” among Segments “2. Concerned” and “4–6. Disengaged–Dismissive.” The agreement level of Segment 2 was as high as that of Segment 1 in the control group for “Desired state,” “Consuming local seafood,” and ”Plastic waste disposal.” The agreement level of Segment 4 in M2 was as high as that of Segment 1 in the control group for “Plastic waste disposal,” and that of Segment 3 in the control group for “Desired state” and “Consuming local seafood.” No significant differences were observed for “Conservation of seaweed beds and tidal flats,” “Conservation of forest,” and “Clean-up beaches and rivers.”

**Fig. 6:**
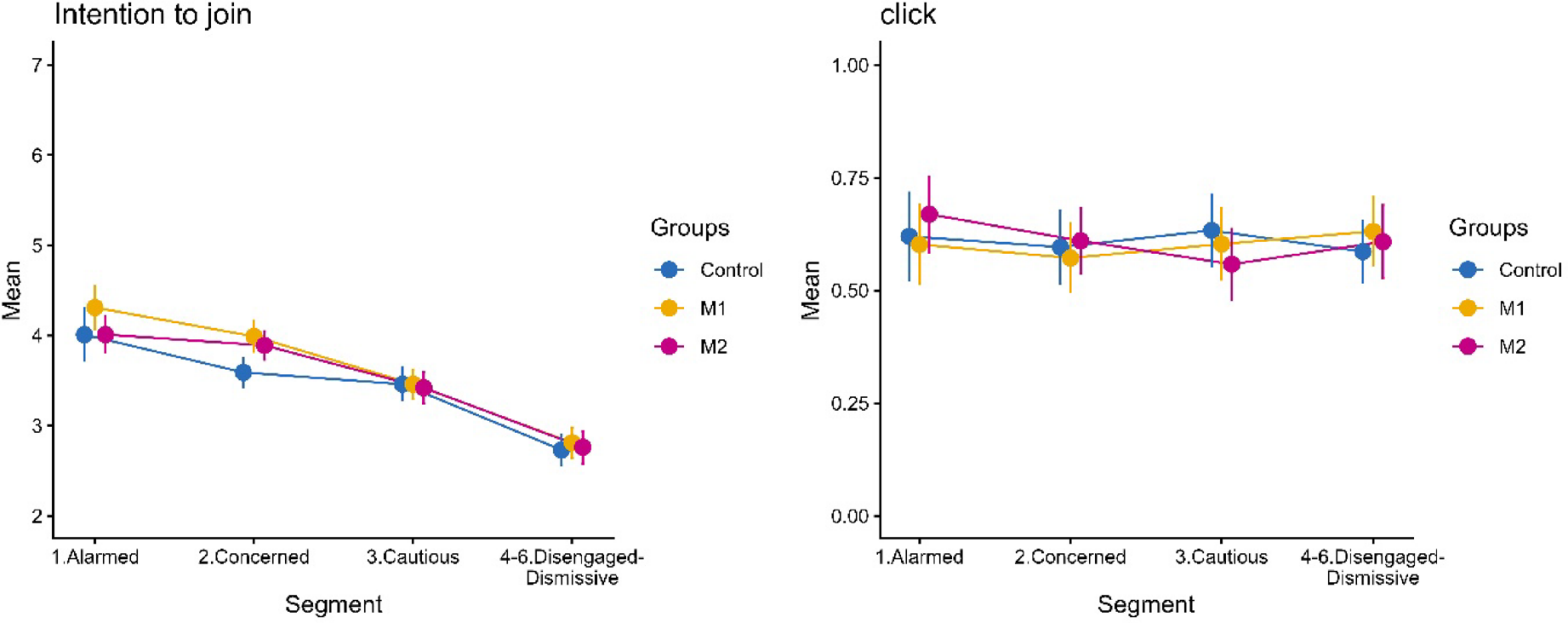
Intention to participate in the “Hyogo Prefectural Citizens’ Council for the Development of an Abundant Sea,” and the click-through rate on the URL of the website by segment and messaging condition. Group 1: Control group, Group 2: M1 (negative consequences of oligotrophication), Group 3: M2 (collective public involvement). Error bars indicate 95% confidence intervals.

Figure 6 shows the intention to participate in the “Hyogo Prefectural Citizens’ Council for the Development of an Abundant Sea” and the click-through rate on the URL for registration for the council. Although significant differences were observed in participation intention by segment, no statistically significant differences were observed by segment in the number of clicks (also see Table 3). Regarding the impact of messaging, only one difference was observed, namely the participation intention among Segment “2. Concerned” in M1 (Negative consequences of oligotrophication, yellow line) was higher than that of the control group. No effect was observed on the click-through rate.

## Discussion

We employed audience segmentation and a messaging approach to investigate the heterogeneity of public understanding of and support for coastal SES management and to elicit practical management implications. Three research questions were posed, namely “RQ1. How different are the sociodemographic characteristics of the public by audience segment concerning their opinions on coastal SES issues and management?”, “RQ2. How different are the levels of support for government-led coastal SES management measures according to audience segments and messaging conditions?”, and “RQ3. How different are attitudes and involvement in coastal SES management according to audience segments and messaging conditions?” We adopted a single-item self-categorization measure for public segmentation^25^. The segments of respondents with negative views about SES and management were small (3.4% of our sample), similar to those of the results of an earlier study on environmental conservation^25^. Therefore, the small segments were merged into “4. Disengaged” and regrouped as “4–6. Disengaged–Dismissive” in the analysis. Our study revealed that the public could be split into four groups, with three being affirmative and one being non-affirmative (i.e., disengaged), along with a limited number of respondents opposing the issue and management.

### Characteristics of the public by audience segment (RQ1)

Audience segmentation revealed the distinctive characteristics of the public, demonstrating the effectiveness of the single-item self-categorization measure used for characterization. The method delineates individuals and the number of people in each segment and informs policymakers on effective targeting of each segment with different attitudes toward and support for coastal SES management. Contrary to the results of earlier studies^25,48^, most characteristics in the current study showed a monotonic relationship (Table 2). Respondents in Segment “Disengaged-Dismissive” tended to be young and physically and psychologically distant from the sea, showed less recognition of problems, and exhibited infrequent related behaviors (e.g., consuming local seafood), with lower values of the sea. Such knowledge helps policymakers effectively target the public in the segment. However, it should also be noted that the audience segmentation did not reveal causal relationships but correlations. Therefore, we cannot say how to move people from one segment to another. Future studies on causal relationships await.

Younger respondents tended to exhibit passive or negative attitudes toward the issue. However, studies on the relationship between age and environmental consciousness did not yield consistent results. Whereas most study results showed stronger consciousness with increasing age^61^, similar to that of our study, some others found an inverse or no relationship^62,63^. These results could be ascribed to the complex effects of various factors, including individual development and the interaction of people with society as they age^62^. The increase in environmentally conscious attitudes and behaviors with age can be partially explained by a psychological shift to a desire to help the next generation and contribute to the future, as outlined in the theory of generativity^61^. However, as this study was based on a survey conducted at a single point in time, it is impossible to determine whether environmental consciousness is an effect of increasing age or generational differences (e.g., the current younger generation is more indifferent than that of the past). However, an effective approach for younger generations is essential, as they are key actors in future SES. Although the long-term effects of the perceived indifference of younger generations require verification, Uehara et al.^40^ found that fish workshops in elementary and junior high schools led to increased understanding and support for coastal SES management for the SIS among the students. Accordingly, concrete and wide-ranging measures for young people should be implemented in cooperation with the education sector.

Physical and psychological distance to the SIS was associated with the segments; physical and psychological distances from the SIS were closest in Segment “Alarmed” and farthest in Segment “Disengaged-Dismissive.” The physical distance finding (travel time in minutes from the SIS to their houses) aligns with that of an earlier study on resident willingness to invest time and effort to improve a coastal area^64^. The psychological distance measured using the INS aligns with that of an earlier finding that psychological closeness correlates with the desire of children to benefit nature^17^. Changing physical distance is difficult; however, psychological distance could be improved, for example, by environmental education which could strengthen human-nature relationships^18,40^. Another approach is to target the public living far from the SIS to inspire their support and involvement.

Problem perception (fishery and oligotrophication) was also inversely related in a monotonic manner, and the difference was statistically significant by segment, indicating that improving public awareness is vital. For both perceptions, the mean values for “Alarmed” were 2.65 (fishery) and 2.67 (oligotrophication) and the mean values for “Disengaged-Dismissive” were 1.48 and 1.42, respectively, indicating a substantial difference in perceptions. Additionally, considering that “5” is the maximum value for “Know well,” even the mean values for “Alarmed” were low; therefore, improving awareness is essential for all segments.

Similar decreasing relationships from “Alarmed” to “Disengaged-Dismissive” were observed for behaviors (visiting the SIS, consuming local seafood, and participating in events related to the SIS), which were all effective in improving attitudes toward SIS problems. Regarding the consumption of local seafood, a large percentage of respondents, particularly in Segment “Disengaged-Dismissive,” chose “3. Don’t know” whether they had consumed local seafood (45.1%). Because one of the reasons for choosing “3. Don’t know” may be that they were not sure if they ate local seafood, efforts to promote the fact that the seafood is local, e.g., collective regional labelling schemes^65^ could also be practical.

Instrumental, relational, and intrinsic values revealed similar decreasing relationships from “Alarmed” to “Disengaged-Dismissive,” and cultivating these values could improve attitudes and support for SES. As values transcend specific contexts, changing them could be difficult^21,66^. However, values could be deep or high-leverage points that could bring about transformational systemic changes^21,67^. Leverage points are places in a system where a small change to them leads to a large systematic shift^67^. Values are one of the deepest leverage points that induce sustainability transformation^21^. A study on an onsite fish workshop found limited but statistically significant impacts of the instrumental, relational, and intrinsic values of the sea on children^40^. While all three values are critical, relational values are particularly promising for sustainability transformation^68^. An empirical study revealed that relational values were closely associated with people’s involvement in coastal SES management^3^.

### Support for government-led coastal SES management measures (RQ2)

Although Uehara et al.^4^ reported an average level of support for government-led coastal SES management measures by the public, we analyzed the public support by audience segment. Support for government-led coastal SES management (i.e., four nutrient supply measures) was overall downward-sloping by segment from most in Segment “Alarmed” to least in Segment “Disengaged-Dismissive” (Fig. 4), indicating that audience segmentation successfully delineated the public in terms of support. For instance, support in Segment “Alarmed” tended to be higher than that in “Disengaged-Dismissive.” The relatively low support level for “FSTP,” reflecting the negative impression of the public on “polluting the sea,” aligned with that of the earlier findings^4^. For example, one respondent stated, “Factory wastewater is massive and quick but may include unwanted substances or be too massive, inversely resulting in eutrophication (Segment “Alarmed,” Control, No. 619).” Following “FSTP,” the support for “Fertilization” was also relatively low. These two measures externally inject nutrients into the seawater, whereas “Seabed plowing” and “Drainage” do not. People could be concerned about unnatural measures, such as FSTP and fertilization. One respondent said, “I think it’s a temporary fix with artificial methods, except for 4 (i.e., “Conservation of seaweed beds and tidal flats”). I think the same problem will continue forever unless it is normalized to something closer to a natural cycle, such as 4 (i.e., “Conservation of seaweed beds and tidal flats”; Segment “Cautious,” M1 (Negative consequences of oligotrophication), No. 595).

Whereas M1 (Negative consequences of oligotrophication) and M2 (Collective public involvement) had some impact on Segment “Disengaged-Dismissive” regarding “Seabed plowing” and “Drainage,” “Cautious” was influenced only by M1 (Negative consequences of oligotrophication) regarding these measures. No impact was observed on “FSTP” and “Fertilization.” As these two segments (i.e., “Cautious” and “Disengaged-Dismissive”) accounted for 50.6% of the public, and overall, the impacts were as high as those of the next segment in the control group, a substantial impact was expected, especially by M1 (Negative consequences of oligotrophication). No impact on measures “FSTP” and “Fertilization” were expected because M1 (Negative consequences of oligotrophication) and M2 (Collective public involvement) did not provide any explanation for removing a negative preconception of “polluting the coastal sea”^4^. Some respondents raised concerns, e.g., “Worried that it may lead to ocean pollution” (Segment “Cautious,” M1 (Negative consequences of oligotrophication), No.968) and “Nutrient supplementation seems artificial and not very impressive” (Segment “Disengaged-Dismissive,” M1 (Negative consequences of oligotrophication), No.1529). Measures “Seabed plowing” and “Drainage” were less likely associated with negative preconceptions. As a respondent stated, “I think it is better to use nature as much as possible, not to do anything artificial” (Segment “Cautious,” M1 (Negative consequences of oligotrophication), No.1155). Therefore, M1 (Negative consequences of oligotrophication), which addresses the severity of the issue, significantly impacted Segments “Cautious” and “Disengaged-Dismissive,” which required more information about the issue. The impact of M2 (Collective public involvement) was limited because it was not related directly to government-led measures. Although not directly comparable because of the use of different messaging content, this finding is consistent with that by Dean et al.^2^. These authors found that moral arguments (M2: Collective public involvement in our study) were less compelling than those of the factual (M1: Negative consequences of oligotrophication in our study) in building community support for coastal management.

However, as no significant impact was observed on “FSTP,” which had relatively low support and involved a negative conception, but was the primary government-led measure, customized messaging focused on the measure in question should be considered to overcome the public’s misconceptions explicitly. For example, messages built on insights gained from the open-ended responses regarding the negative conception could effectively address those concerns. Furthermore, both types of messages were conveyed using specific poster designs, and even if the purpose was the same, different designs could have varying effects. For future studies, the effectiveness of other messaging conditions built on other approaches, such as community-based social marketing^24^, may be worth testing.

### Attitudes and involvement in coastal SES management (RQ3)

The attitudes and involvement of the public, both in terms of general intention to contribute and individual items, differed monotonically by the audience segment (i.e., downward sloping, as shown in Fig. 6), implying that the more affirmative they were about SIS issues and taking action, the more they agreed.

Regarding individual items, support for the idea of a “Desirable state” and “Plastic waste disposal” tended to be high, whereas that for “Conservation of seaweed beds and tidal flats,” “Conservation of forests,” and “Cleanup beaches and rivers” was generally low, and that for “Consuming local seafood” was intermediate. One probable explanation is that they require specific actions for environmental conservation, whereas “Desirable state” supports the notion without requiring actions^4^. According to some respondents, “I know people are all very environmentally conscious, but they do not have the time” (Segment “Disengaged-Dismissive,” M2 (Collective public involvement), No. 1508) and “Low-income households that cannot afford the lifestyle of being able to work as volunteers” (Segment “Disengaged-Dismissive,” M2 (Collective public involvement), No.1032). Support for “Plastic waste disposal” was high, and in addition to creating a rich ocean, this measure is associated broadly with the plastic waste problem that society as a whole is trying to resolve. Furthermore, the public broadly recognizes this issue. The hurdle for taking action on “Consuming local seafood” was lower than those in “Conservation of seaweed beds and tidal flats,” “Conservation of forests,” and “Cleanup beaches and rivers” because acting in this instance could be easier. However, the availability of local seafood and differences in preferences for fish could hinder action. One respondent said, “Prices are too high to consume local seafood” (Segment “Disengaged-Dismissive,” M2 (Collective public involvement), No.164).

The impact of messaging conditions varied between M1 (Negative consequences of oligotrophication) and M2 (Collective public involvement). Whereas M1 (Negative consequences of oligotrophication) impacted Segments “Cautious” for “Desired state” and “Disengaged-Dismissive” for “Desired State” and “Plastic waste disposal,” M2 (Collective public involvement) impacted Segments “Concerned” and “Disengaged-Dismissive” for “Desired state”; “Consuming local seafood”; and “Plastic waste disposal. The impact of M2 (Collective public involvement) on “Consuming local seafood” is reasonable, as clearly indicated in the message (Fig. 2b). The impacts of both messaging conditions on Segment “Disengaged-Dismissive” indicated that they appealed to the public who were indifferent or negative about this issue. For example, the agreement level of Segment “Disengaged-Dismissive” became as high as that of Segment “Alarmed” for “Plastic waste disposal.” If Segment “Disengaged-Dismissive” is the primary target, M2 (Collective public involvement) is preferable because it has a broader and more profound impact on attitude and involvement. It should also be noted that neither M1 (Negative consequences of oligotrophication) nor M2 (Collective public involvement) had an impact on any segment for the categories of “Conservation of seaweed beds and tidal flats,” “Conservation of forests,” and “Cleanup beaches and rivers,” highlighting that a message alone is insufficient to influence the likelihood of more challenging behaviors.

The intention to participate in the “Hyogo Prefectural Citizens’ Council for the Development of an Abundant Sea” showed similar differences across the segments. However, the effect of messaging was limited. No effect was observed on Segments “Cautious” and “Disengaged-Dismissive,” which was observed in the support for nutrient supply measures, attitudes, and involvement in coastal SES management. As participation in the council requires more direct involvement than simply showing support for government-led measures and changing one’s everyday life behaviors, such as consuming local seafood and properly disposing of plastic waste, this was difficult to change.

No differences were observed in the click-through rates that can be viewed as an indicator of actual involvement behavior, as opposed to intentions^25^. However, studies applying this method to environmental issues produced mixed results. Naito et al.^25^ found some effect of messaging, whereas Blake et al.^69^ found differences by country but no differences by segment or the type of message supplied. Respondents could click the URL only out of curiosity and not to sign up for the council. Further studies are required to determine whether this measurement method is appropriate as a proxy for behavior.

## Conclusion

We employed audience segmentation to investigate the importance of elucidating the heterogeneity of public support and involvement in coastal SES management. The segmentation successfully identified distinct sociodemographic characteristics and revealed varying support for government-led coastal SES measures (i.e., nutrient supply measures) and attitudes and involvement in coastal SES management. The levels of support for government-led coastal SES measures and attitudes toward and involvement in coastal SES management sloped downward. Furthermore, the two messaging conditions (“Negative consequences of oligotrophication” and “Collective public involvement”) demonstrated varying effects on support, attitudes, and involvement by segment.

This study makes three major academic and practical contributions to the literature. First, we demonstrated the effectiveness of audience segmentation in capturing public heterogeneity. Such information helps policymakers effectively target specific segments (e.g., people disinterested in the issue). Our findings are based on a case study of a coastal SES but could generally be informative for researchers studying SESs, i.e., not restricted to coastal areas. Second, messaging conditions provided insights into measures for approaching targeted segments. Third, we demonstrated the usefulness of the single-item self-categorization measure. The application of this measure has been limited in sustainability science^25,29,48,49^.

This study had six limitations. First, we have merged “4. Disengaged,” “5. Doubtful,” and “6. Dismissive” into a single segment “4–6. Disengaged–Dismissive” owing to small sample sizes, resulting in a loss of variability in the respondents^25^. It is critical to test a J-shaped curvilinear relation on outcomes related to engagement in the topic^49^. A larger sample size is expected in future studies. Second, although our single-item self-categorization assessment successfully segmented the public, we did not assess the test–retest reliability of the items^49^, as this was outside our study scope. However, developing items applicable to other types of SESs is crucial. Third, because our study measures message effects using a single-point survey, the duration of these effects remains unknown. Fourth, the support for the government-led coastal SES measures and attitudes toward and involvement in coastal SES management could be related as complements or substitutes^70^. Government-led coastal SES measures could discourage collective public involvement if they are perceived as substitutes. Future studies on this point await. Fifth, click-through rates should be reconsidered as a proxy for actual behavior, as we did not observe differences by segment. An alternative is to measure the actual behaviors. For example, the number of respondents who actually signed up for the Council could be counted by coordinating the survey website and the sign-up website. After implementing the survey, the number of individuals registering for the council doubled (email communication with a staff member of the Hyogo Prefectural Government on March 3, 2025). However, we cannot confirm whether these people were registered in our survey in the first place. They can be traced by asking related questions when they register with the council. Lastly, more messaging conditions should be tested to find an effective message for the targeted public regarding the targeted issue.

## Supplementary Information

Below is the link to the electronic supplementary material.

Supplementary Material 1

Supplementary Material 2

Supplementary Material 3

## Data Availability

All data are available as supplementary material (SM2) for replicability.
