# Supplementary material for: Audience segmentation and messaging approach to gain public support and involvement in coastal social-ecological system management
Source: Sci Rep. 2026 Feb 3;16:7025. doi: 10.1038/s41598-026-38402-0 (PMC12921029; doi:10.1038/s41598-026-38402-0)
Supplement: Supplementary file 1 — Supplementary Material 1 [file 41598_2026_38402_MOESM1_ESM.docx]

Supplementary Material 1 (SM1)

Table A1. Age and sex distribution of participants

|  |  | Participants | | Population* |
| --- | --- | --- | --- | --- |
| Sex** | Age | Frequency | % | % |
| Male | 20–29 | 63 | 7.3% | 13% |
|  | 30–39 | 162 | 18.8% | 14% |
|  | 40–49 | 156 | 18.1% | 18% |
|  | 50–59 | 129 | 14.9% | 17% |
|  | 60 and above | 354 | 41.0% | 38% |
|  | Sub total | 864 | 100.0% | 100% |
| Female | 20–29 | 106 | 11.3% | 11% |
|  | 30–39 | 137 | 14.6% | 13% |
|  | 40–49 | 168 | 17.9% | 16% |
|  | 50–59 | 144 | 15.4% | 16% |
|  | 60 and above | 381 | 40.7% | 44% |
|  | Sub total | 936 | 100.0% | 100% |
|  | Total | 1800 |  |  |

*Hyogo Prefectural Government. There is no “Other” category, including intersex, in the official statistics.

**No respondent chose “Other.”

Table A2. Composition of respondents by segment and control-treatment group

| Segment | | Alarmed | Concerned | Cautious | Disengaged | Doubtful | Dismissive | Total |
| --- | --- | --- | --- | --- | --- | --- | --- | --- |
| Pooled  (n = 1,800) | | 19.3% | 27.2% | 25.2% | 25.0% | 1.2% | 2.2% | 100% |
|  | Control  (n = 600) | 16.7% | 24.3% | 24.5% | 26.7% | 1.8% | 6.0% | 100% |
|  | M1  (n = 600) | 20.5% | 27.7% | 25.2% | 25.0% | 1.2% | 0.5% | 100% |
|  | M2  (n = 600) | 20.7% | 29.5% | 26.0% | 23.3% | 0.5% | 0.0% | 100% |
