# Supplementary material for: Audience segmentation and messaging approach to gain public support and involvement in coastal social-ecological system management
Source: Sci Rep. 2026 Feb 3;16:7025. doi: 10.1038/s41598-026-38402-0 (PMC12921029; doi:10.1038/s41598-026-38402-0)
Supplement: Supplementary file 3 — Supplementary Material 3 [file 41598_2026_38402_MOESM3_ESM.pdf]

Supplementary Material 3 (SM3): Questionnaire

*This is a translation from the survey in Japanese

Part 1: Basic characteristics of respondents

1) What is your age?

[ ] years old

2) What is your gender?

1) Male
2) Female
3) Other

What is your highest level of education?

1) Middle School
2) High School
3) Vocational school
4) University
5) Graduate school

In which are do you live?

1) Kobe-Hanshin Region
2) Harima Region

How long does it take to get to the SIS? Think about the transportation method that you use probably the most.

[ ] minutes

Choose the figure which captures your relationship with the SIS the most.

Relationship 1 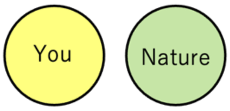

Relationship 2 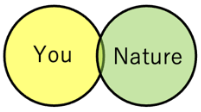

Relationship 3 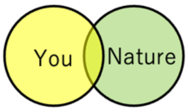

Relationship 4 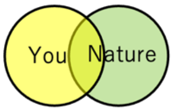

Relationship 5 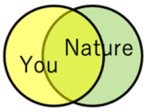

Relationship 6 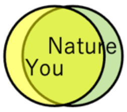

Relationship 7 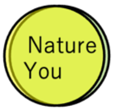

Do you know that some fish are no longer caught, and seaweed is discolored in the Seto Inland Sea?

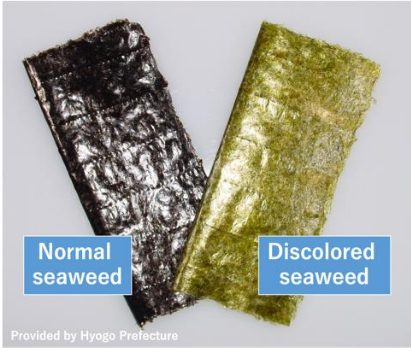

1) I have never heard of it
2) I have heard of it, but do not know about it
3) I know a little
4) I roughly know about it
5) I know it well

Did you know that some areas of the SIS lack the nutrients necessary for living organisms?

1) I have never heard of it
2) I have heard of it, but do not know about it
3) I know a little
4) I roughly know about it
5) I know it well

How often do you have the opportunity to visit the SIS?

1) Rarely
2) Once every few years
3) A few times a year
4) Once a month
5) More than once a week

Have you eaten fish from the Seto Inland Sea at home in the past week?

1) Yes
2) No
3) I don’t know

Did you participate in any ocean-related events held in Hyogo Prefecture last year (2024)? Events included, for example, the “Hyogo Abundant Sea Development Promotion Convention,” “Fish Culture Festival Akashi,” and the "Suma Abundant Sea Project.

1) Yes
2) No
3) I don’t know

The following are some thoughts and opinions about the SIS and its blessings. On a scale of 1 to 5, from “Strongly disagree” to “Strongly agree”, please select the one that best reflects your opinion.

	Strongly disagree	Disagree	Neither	Agree	Strongly agree

The SIS is important to me, because it provides food, such as seafood.	1	2	3	4	5
The marine ecosystem of the SIS is important to me as a means for preventing and mitigating disasters, such as floods, tsunamis, and typhoons.	1	2	3	4	5
The marine ecosystem of the SIS is important to me because it purifies the seawater.	1	2	3	4	5
The SIS is important to me, because it maintains the food chain (the relationship between animals and plans of eating and being eaten).	1	2	3	4	5

The following are some thoughts and opinions about our relationship with the SIS. On a scale of 1 to 5, from “Strongly disagree” to “Strongly agree”, please select the one that best reflects your opinion.

	Strongly disagree	Disagree	Neither	Agree	Strongly agree
The SIS is an important location for me	1	2	3	4	5
The SIS is an important location for local residents.	1	2	3	4	5
I am able to connect with others through my relationship to the SIS.	1	2	3	4	5
Caring for the SIS leads to caring for the people of the present and future.	1	2	3	4	5
We have a moral responsibility to protect the SIS and its creatures.	1	2	3	4	5
Protecting the SIS fills me with a sense of contentment and enables me to lead a good life.	1	2	3	4	5

Maintaining the SIS in good condition is the right thing to do.	1	2	3	4	5

The following are some thoughts and opinions about the SIS. On a scale of 1 to 5, from “Strongly disagree” to “Strongly agree”, please select the one that best reflects your opinion.

	Strongly disagree	Disagree	Neither	Agree	Strongly agree
Every living organism in the Seto Inland Sea has the right to live.	1	2	3	4	5
The Seto Inland Sea should be protected for nature itself, regardless of whether it is for us or not.	1	2	3	4	5

Part 2: Opinions about nutrient supply

To make the SIS rich a sea, the Hyogo Prefectural Government increases the supply of nitrogen and phosphorus (nutrients) to combat oligotrophication (a condition in which there is not enough nutrients).

Which of the following statements do you most agree with?

1) Alarmed: I am very concerned about the oligotrophication of the SIS and I believe that governments and individuals should take immediate action.

2) Concerned: I am concerned about the oligotrophication of the SIS and believe that action must be taken although there is still time to determine an appropriate response.

3) Cautious: I think the SIS is becoming oligotrophic, although I am not sure. We must carefully determine the timing of and mechanism for interventions.

4) Disengaged: I have not thought much about the SIS becoming oligotrophic.

5) Doubtful: I do not think the SIS has become oligotrophic, although I am not certain. I am more concerned about an overreaction to the assumed oligotrophication.

6) Dismissive: I do not think neither oligotrophication of the SIS is occurring nor that humans have caused it. Therefore, I am neither inclined to take action to address oligotrophication nor do I support such action.

Note:

Posters were presented only to those who were in the treatment groups.

[Messaging Condition 1: Negative consequences of the oligotrophication]

Please read the poster carefully. After the poster, there will be a comprehension quiz. If your answer is incorrect, your answer will be automatically terminated.

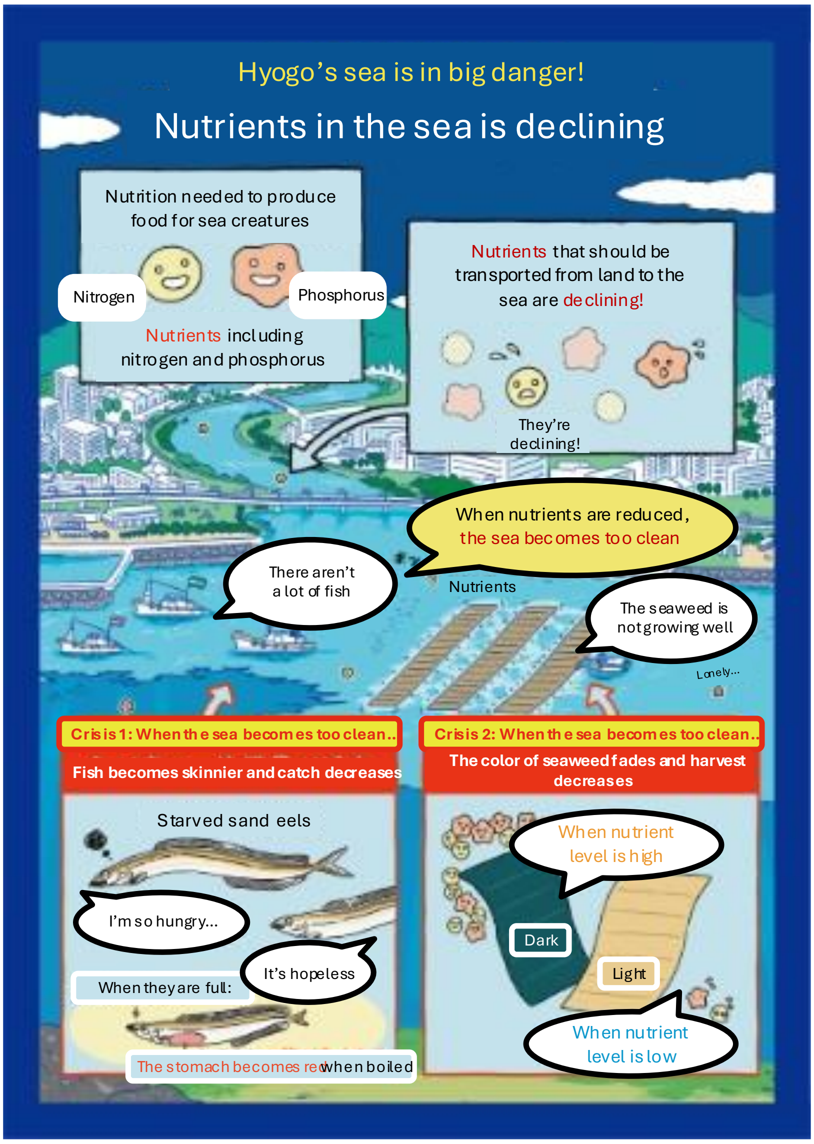

Please select all the correct statements from the previous poster.

1) Hyogo's seas are rich in nitrogen and phosphorus

2) Fish are becoming thinner and catches are decreasing
3) Nori is losing color and harvests are decreasing

[Messaging Condition 2: Collective public involvement in the coastal SES management]

Please read the poster carefully. After the poster, there will be a comprehension quiz. If your answer is incorrect, your answer will be automatically terminated.

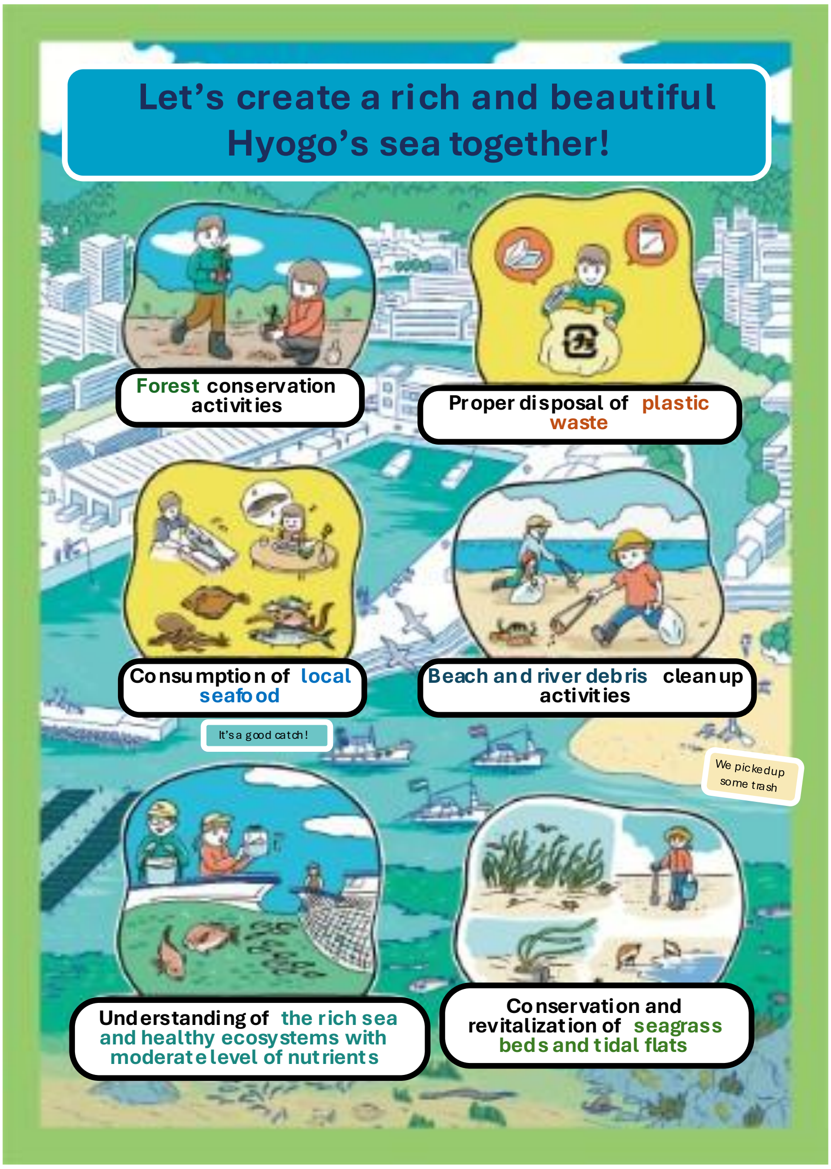

Please select all correct statements from the previous poster.

1) Hyogo's seas are rich in nitrogen and phosphorus
2) Coastal debris and river cleanup activities are important
3) A rich ocean with moderate nutrition and a healthy ecosystem must be understood

6) On a scale of “1. Completely not supportive” to “6. Completely supportive,” the following four measures to manage the supply of nutrients implemented by Hyogo Prefecture, please choose from the following six options.

1) Nutrient supply from factories and sewage treatment plants

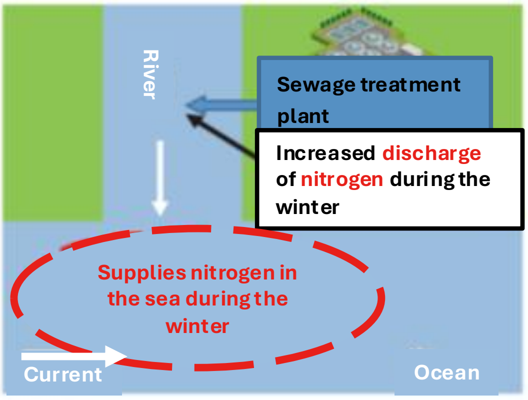

2) Fertilization of the sea

Note to readers: The image of the sea fertilization was removed from the supplementary material because it includes images that could lead to identification of the people involved.

Direct input of organic fertilizers and other materials into the sea

3) Seabed plowing

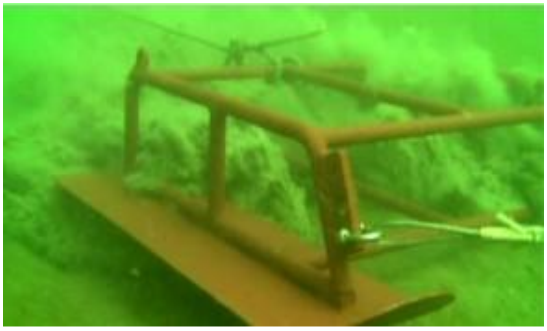

Plowing the seafloor and eluting accumulated nutrients into the sea

4) Nutrient supplies from reservoirs and forests

Note to readers: The image of the nutrient supplies from reservoirs and forests was removed from the supplementary material because it includes images that could lead to identification of the people involved.

Water and mud from reservoirs rich with nutrients are discharged into the sea

	Completely not supportive	Not supportive	Rather not supportive	Neither	Supportive	Rather supportive	Completely supportive

1. Supplying nutrients from factories and sewers	1	2	3	4	5	6	7
2. Fertilizing the sea	1	2	3	4	5	6	7
3. Plowing the seabed to disperse the nutrients on the seafloor	1	2	3	4	5	6	7
4. Discharging nutrients from forests into the sea	1	2	3	4	5	6	7

Please write down any impressions or thoughts you may have about each of the above measures.

【 】

Do you want to contribute to making the Seto Inland Sea a richer place?

1) Strongly disagree
2) Disagree
3) Neither agree nor disagree
4) Agree

The following can be thoughts and actions regarding the management of the Seto Inland Sea. On a scale of 1 to 5, from “Strongly disagree” to “Strongly agree”, please select one that best reflects your opinion.

	Strongly disagree	Disagree	Neither	Agree	Strongly agree
The Seto Inland Sea should aim to become a “rich sea” with a balance between water quality (transparency) and fish catch.	1	2	3	4	5
I would like to participate in sea-related events held in the coastal zone of the Seto Inland Sea and visit the aquariums and beaches.	1	2	3	4	5
If I am consuming seafood, I would like to eat seafood from the Seto Inland Sea.	1	2	3	4	5
I try to dispose of my plastic waste correctly.	1	2	3	4	5
I want to contribute to the preservation activities of the Seto Inland Sea.	1	2	3	4	5
I want to contribute to cleanup activities in the Seto Inland Sea.	1	2	3	4	5

7) Please describe any obstacles to the participation of prefectural residents in the creation of an abundant ocean.

【　　　　　　　　　　　　　　　　　　　　　　　】

Part 3: Behaviors related to nutrient supply

8) Hyogo Prefectural Government has established the the “Hyogo Prefectural Citizens' Council for the Development of an Abundant Sea” to energize the citizens of the prefecture in their efforts to create an abundant sea, and is engaged in the dissemination of information through workshops, the holding of events that can be enjoyed by all, and

networking among members. You can join as an individual. Membership is free. To what extent may I join?

1) Will participate, of course
2) Very likely to participate
3) Will probably participate
4) Undecided
5) Probably will not participate
6) Very unlikely to participate
7) Definitely will not participate

-Already a member

If you wish to become a member, please click “Hyogo Prefectural Citizens' Council for the Development of the Hyogo Plentiful Sea” below. The website of Hyogo Prefecture's “Hyogo Prefecture Citizens' Council for the Development of the Hyogo Plentiful Sea” will open in a new window. Please note that no personal information that can identify you will be collected by the survey administrator as a result of your click. Nor will it affect your compensation for answering this survey.

ひょうご豊かな海づくり県民会議
